# Effect of using client-accessible youth health records on experienced autonomy among parents and adolescents in preventive child healthcare and youth care: A mixed methods intervention study

**DOI:** 10.1177/13674935231177782

**Published:** 2023-05-25

**Authors:** Janine Benjamins, Emely de Vet, Gerlinde Jordaan, Annemien Haveman-Nies

**Affiliations:** 1 Consumption and Healthy Lifestyles, 4508 Wageningen University & Research, Wageningen, Netherlands; 2 Icare JGZ, Blankenstein 550 , Meppel, Netherlands; 3 GGD NOG 4508 , Warnsveld, Netherlands

**Keywords:** electronic health records, child health services, personal autonomy, professional role, child welfare

## Abstract

Client autonomy is important in Dutch youth care. It correlates positively with mental and physical health and can be strengthened by professional autonomy-supportive behaviour. Aiming for client autonomy, three youth care organisations co-developed a client-accessible youth health record (EPR-Youth). Currently, limited research is available on how client-accessible records contribute to adolescent autonomy. We investigated whether EPR-Youth strengthened client autonomy and whether professional autonomy-supportive behaviour reinforced this effect. A mixed methods design combined baseline and follow-up questionnaires with focus group interviews. Different client groups completed questionnaires about autonomy at baseline (*n* = 1404) and after 12 months (*n* = 1003). Professionals completed questionnaires about autonomy-supportive behaviour at baseline (*n* = 100, 82%), after 5 months (*n* = 57, 57%) and after 24 months (*n* = 110, 89%). After 14 months, focus group interviews were conducted with clients (*n* = 12) and professionals (*n* = 12). Findings show that clients using EPR-Youth experienced more autonomy than non-users. this effect was stronger among adolescents aged 16 and older than among younger adolescents. Professional autonomy-supporting behaviour did not change over time. However, clients reported that professional autonomy-supporting behaviour contributed to client autonomy, emphasising that professional attitude needs addressing during implementation of client-accessible records. Follow-up research with paired data needs to strengthen the association between using client-accessible records and autonomy.

## Introduction

### Autonomy

Personal autonomy is highly valued in the Western world (Kara, 2007; Chirkov, 2008). In line with self-determination theory, autonomy is conceived as ‘acting in accordance with someone’s intrinsic motivation’ and ‘making choices that contribute to a life which is valued as good’ (Chirkov et al., 2003; Meinema, 2017). In Western medicine and medical ethics, respect for patient autonomy has become a basic principle (Varelius, 2006). Patient autonomy means that patients have a right to make informed decisions about their medical care without healthcare providers trying to steer their decisions (Varelius, 2006). Involving patients in their own care positively correlates with mental and physical health and higher levels of health behaviour (Ng et al., 2012).

### Professional autonomy-supportive behaviour

**Figure 1.**
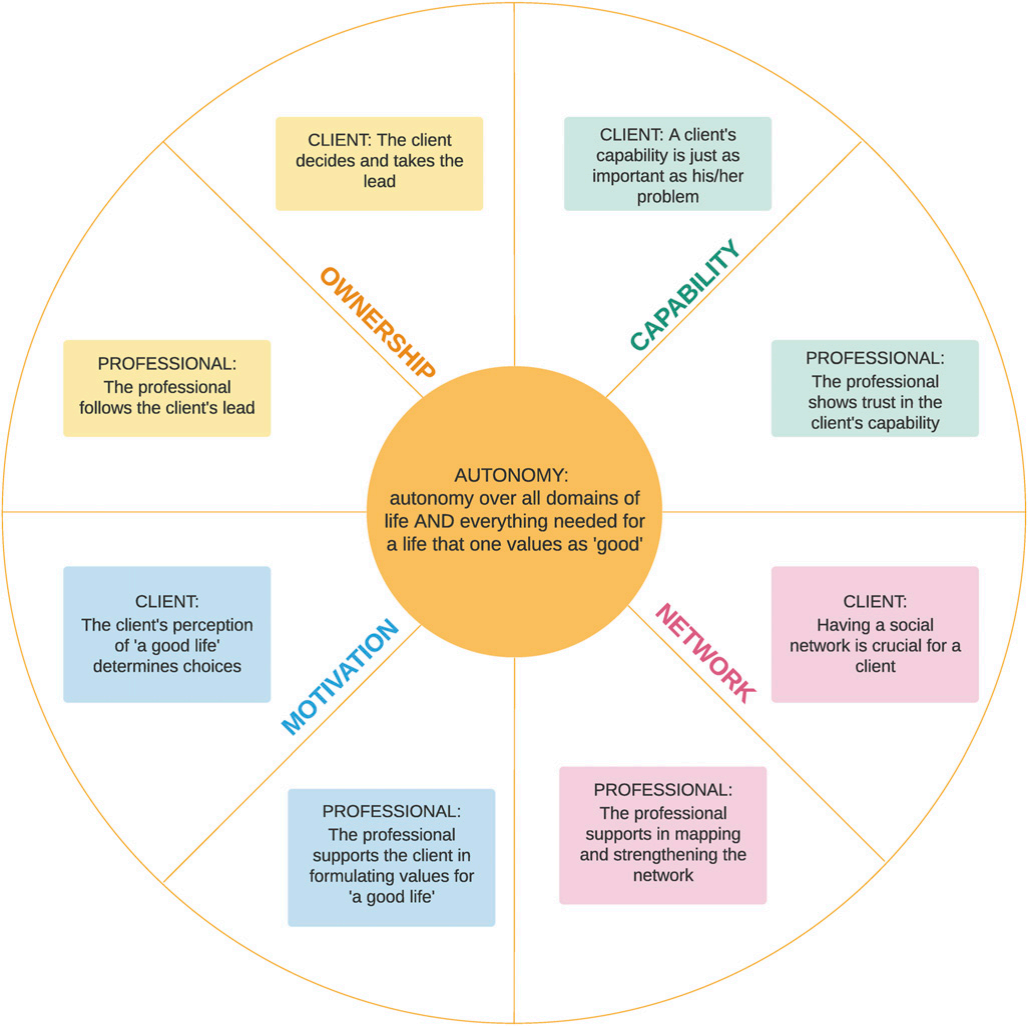
How to contribute to client’s autonomy, (Meinema, 2017).

Professionals can strengthen patient autonomy with autonomy-supportive behaviour, characterised by affirming patients’ ownership of their health decisions, following their motivation, showing confidence in their capability, and supporting them to strengthen their network (Figure 1) (Entwistle et al., 2010; Meinema, 2017).

### Autonomy in Dutch youth care

In the Netherlands, since the new Youth Act was introduced in 2015, enhancing client autonomy has been prioritised in ‘care for youth’, initiating a paradigm shift from professional-centred care towards client- and family-centred care (Dutch Ministry of Public Health, 2013, 2014). Consequently, to enhance client autonomy, three Dutch organisations delivering youth care or preventive child healthcare (PCH) cooperatively developed a client-accessible electronic patient record for youth (EPR-Youth), accessible to parents of children aged up to 16 years and adolescents aged 12 years and older (Benjamins et al., 2018).

### Challenges for autonomy among adolescents

In the field of child and adolescent health and well-being, using client-accessible health records to aim for autonomy raises two challenges. First, adolescents’ increasing independence has changed a parent’s role. Until the age of approximately 12, parents are responsible for their childrens’ upbringing and development; they exercise autonomy on their childrens’ behalf (United Nations, 1989a). However, when adolescents get older, they increasingly gain autonomy over their life and health decisions, and client autonomy becomes a shared domain between parents and adolescents (Dutch Ministry for Justice, 2006). Second, parents and adolescents both have a right to access the adolescent’s records and a right to privacy (Dutch Ministry for Justice, 2006; United Nations, 1989b).

### Gap in knowledge

These challenges have globally hindered the development of client-accessible records for adolescents (Bayer et al., 2015; Bourgeois et al., 2018; Sarabu et al., 2021). Consequently, little is known about the role of client-accessible records in enhancing autonomy in an age group that is transitioning to adulthood. Research in adult healthcare shows that transparent patient-accessible records make patients feel better informed and more engaged in their own care, which contributes to patient autonomy. (Benjamins et al., 2021; Davis Giardina et al., 2014; Mold et al., 2015). Whether the same applies for adolescents yet needs addressing.

### Aim

To investigate whether using EPR-Youth in youth care and PCH contributed to experienced autonomy among adolescents and parents and whether professional autonomy-supportive behaviour enhanced the effect of using EPR-Youth.

## Methods

### Intervention EPR-Youth

EPR-Youth has been built for six municipalities in the North Veluwe region. It facilitates all professionals working in the regional Centres for Youth and Family (CJGs) (Benjamins et al., 2018); it comprises three different organisations, one providing PCH to preschool children (PCH 0–3), one providing PCH to school-children and adolescents (PCH 4–18) and one providing youth care. The CJGs provide preventive healthcare to all 39,560 children aged up to 18 years in the region, and additional youth care to children with behavioural or sociopsychological problems (North-Veluwe Region, 2017; Dutch Bureau for Statistics, 2022).

EPR-Youth has a tethered client portal in which parents and adolescents can read everything professionals register. They can manage appointments, ask questions and write comments. Complying with Dutch legislation, adolescents get portal access after turning 12 (Dutch Ministry of Justice, 2006). Parental access is revoked after a child turns 16 unless rejected earlier by an adolescent aged 12 years or older. Furthermore, adolescents between 12–16 years of age can keep specific information confidential between themselves and a professional.

When EPR-Youth was first introduced in September 2019, a client-accessible EPR was new for most parents and adolescents. Parents of preschool children, however, were already acquainted with a client portal offering limited insight and planning functions.

### Research design

A mixed methods research design with an explanatory sequential approach was chosen. Questionnaires were conducted at baseline (prior to introducing EPR-Youth), followed by one follow-up questionnaire among parents and adolescents, and two follow-up questionnaires among professionals. Two months after completing both client questionnaires, focus group interviews were conducted with representatives of all three target groups. Data were collected between November 2018 and September 2021.

### Study population and inclusion

The study included three groups in the North Veluwe region: parents of children aged up to 16 years, adolescents aged 12 years and older, and professionals working in the three CJG organisations. Different samples of parents and adolescents were invited to complete an online questionnaire when visiting a CJG at baseline (T0) or 12 months after introducing EPR-Youth (T1) (Supplementary Material 1). All CJG professionals were invited at baseline (T0) to complete an online questionnaire; all responders received a link to a follow-up questionnaire 5 months later (T1). Because of low response at T1, we broadened the scope for the second follow-up questionnaire after 24 months (T2) and re-invited all professionals.

All CJG professionals were invited for focus group interviews, along with clients who had completed a questionnaire. From those who wanted to participate, two groups of clients and professionals, respectively, were selected through purposive sampling.

### Questionnaires

Two different questionnaires were developed for clients and professionals. Both addressed socio-demographic characteristics and elements of client autonomy: ownership, motivation, capability, and, among professionals, networks. These elements were derived from a Dutch model, describing what each element meant from a client’s perspective and which professional autonomy-supportive behaviour this was associated with (Figure 1) (Meinema, 2017).

### Socio-demographic characteristics

The clients’ questionnaire contained questions about age, sex, educational level, native country and family composition because previous findings show that these characteristics might influence portal use (Benjamins et al., 2021; Hoogenbosch et al., 2018; Wildenbos et al., 2017). Due to a flaw in our online questionnaire instrument, the variable ‘native country’ was only collected in the follow-up measurement. Questions were equal for parents and adolescents, excluding answering categories for educational level (Supplementary Material 1). For parents, educational level was classified into three categories based on the Dutch Standard Classification of Education (Dutch Bureau for Statistics, 2021). For adolescents, an adapted classification was used with two categories. Parents and adolescents were asked which CJG organisation they had visited because of the possible differences in autonomy-supportive behaviour between professionals from different organisations. Both groups were asked if they used the client portal. The professionals’ questionnaire contained questions about sex, organisation, profession and working experience, because of a possible influence on attitudes towards using EPR-Youth.

### Experienced client autonomy

Experienced autonomy was measured with five items representing three elements of autonomy (ownership, motivation and capability). Adolescents and parents were asked to rate on a 5-point Likert scale ranging from 1 (very negative/never) to 5 (very positive/always) to what extent they experienced they could ‘choose a plan and solution that fits with you and your family’ (ownership), received ‘advice that matches your needs’ (motivation), or felt encouraged to ‘build further on things you already know, capacities you have and things you already do’ (capability) (Supplementary Material 1). A self-constructed questionnaire was developed and tested for content validity with experts on Patient Reported Experience Measures, and with professionals.

Using Maximum Likelihood extraction factor analysis, one factor could be extracted with Eigenvalues above 1.0, explaining 54.9% of all variance (α = 0.77). Consequently, we created individual composite scores, calculating individual mean ‘autonomy’ scores when at least three out of five questions were completed.

### Professional autonomy-supportive behaviour

To measure a professional’s degree of autonomy-supportive behaviour, we operationalised the autonomy model (Figure 1) into questions about ‘capability’ (e.g. whether professionals ask clients what is going well), ‘network’ (e.g. whether professionals ask who else is concerned with a client’s well-being), ‘motivation’ (e.g. whether professionals explores a clients’ values for ‘a good life’) and ‘ownership’ (e.g. whether professionals let clients decide what they want to keep and what needs to change) (Supplementary Material 2) (Meinema, 2017). Professionals reported on a 5-point Likert scale, ranking from 1 (‘always’ or ‘totally agree’) to 5 (‘never’ or ‘totally disagree’).

Using a Maximum Likelihood factor analysis, one factor was extracted, explaining 42.2% of the variance (α = 0.7). Therefore, individual composite scores were created, calculating individual mean ‘autonomy-supportive behaviour’ scores when at least six out of eight questions were completed.

### Focus group interviews

To prevent group bias, an independent and experienced moderator conducted all focus group interviews with clients and with professionals. A semi-structured questionnaire (Supplementary Material 3) guided the interviews, addressing how participants experienced using EPR-Youth contributed to client autonomy, and whether client-professional interaction affected either portal use or client autonomy. An observer assisted the moderator in ensuring that all topics were discussed. To ensure confidentiality, quotes from focus group participants have been pseudonymised in this manuscript.

### Statistical analysis

IBM SPSS Statistics 27.0 was used to analyse quantitative data. Descriptive statistics were used to describe participants’ socio-demographic characteristics. Assumptions for the parametric tests were tested, and none were violated. Differences in respondents’ socio-demographic characteristics between baseline and follow-up were tested using Pearson’s Chi-square tests for both professionals and clients. Data from parents and adolescents were analysed separately.

### Experienced client autonomy

A linear regression model was used to analyse differences in client autonomy scores between baseline and follow-up. Initially, educational level, sex, native country, family composition, portal use, organisation, and differences between baseline and follow-up were included in the model, as well as relevant interactions between those variables. After backward elimination, for parents, differences between baseline and follow-up, portal use and organisation were included with fixed main effects, and interaction between the first two variables was included. For adolescents, differences between baseline and follow-up and age, and interaction between them, were included with fixed main effects.

### Professional autonomy-supportive behaviour

Changes in professional autonomy-supportive behaviour were analysed using a repeated-measures analysis of variance (ANOVA) over all paired data. Initially, work experience, organisation, profession and sex were included in the model as fixed factors. After backward elimination, all factors were excluded. To optimise data use, changes in professional contribution to autonomy were also tested using unpaired data of all professionals participating in T0 and T2. We compared all responders at T0 with those who only completed T2 and all responders at T2 with those who only completed T0, using a linear regression model, including organisation with fixed main effect.

### Qualitative data analysis

The qualitative data were recorded and transcribed verbatim, and a member check was conducted with all participants to confirm transcript accuracy. Data were analysed using ATLAS.ti, versions 8 and 9. Three researchers (JB, AB and GJ) performed a thematic analysis based on the Movisie model (Meinema, 2017) (Figure 1). Two independent researchers coded each interview transcript combining inductive and deductive coding. Differences in coding were iteratively discussed between coding researchers, and themes were generated. Subsequently, theme interpretation was discussed with all authors, and minor modifications were made.

### Data integration

Connecting, building and merging were used to integrate all data through a narrative approach (Fetters et al., 2013). Questionnaire respondents were recruited to participate in focus groups (connecting), and the focus group interview guide informed the questionnaire outcomes (building). The outcomes from both quantitative and qualitative analyses were combined and compared (merging) to reach conclusions.

### Ethical approval and consent to participate

All methods were carried out according to the relevant guidelines and regulations of the Netherlands Code of Conduct for Scientific Practice. The research protocol was approved by the Social Sciences Ethics Committee of Wageningen University, approval number 2018-24-Benjamins.

All questionnaire respondents and focus group participants received study information and gave their consent before participation. For minor participants, consent to participate was given by themselves and their parents/guardians.

## Results and findings

### General characteristics

At baseline, 1202 parents and 202 adolescents completed a questionnaire. A different group of 914 parents and 89 adolescents completed the follow-up questionnaire after 12 months. Completing socio-demographic questions was non-mandatory leading to missing data for different characteristics, which we considered missing at random. Client respondents at baseline and follow-up differed significantly for all characteristics, excluding sex distribution among parents (Supplementary Material 4). Native country was only measured during follow-up. Compared with the source population, parents completing the questionnaire were more often women, highly educated or native Dutch (Dutch Bureau for Statistics, 2022). Among adolescents, native Dutch respondents were overrepresented (Dutch Bureau for Statistics, 2022).

**Figure 2.**
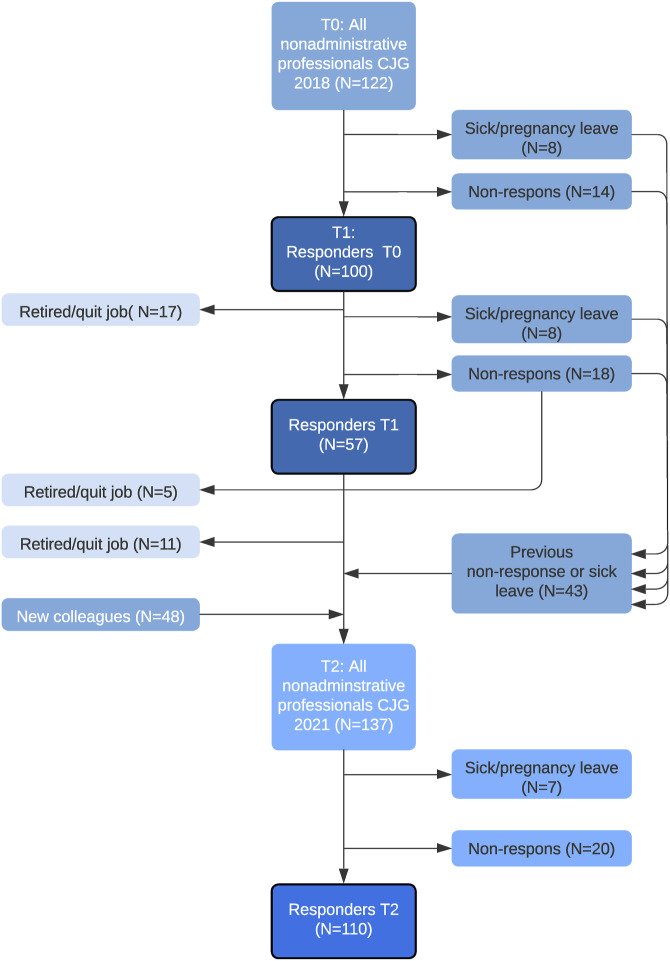
Flow chart inclusion and response professional questionnaire.

At baseline, 100 (82%) out of 122 invited professionals completed the professional questionnaire, 57 (57%) of the baseline responders completed the first follow-up questionnaire and 122 (89%) out of 137 invited professionals completed the second follow-up questionnaire (Figure 2). Professional respondents’ characteristics did not differ significantly between T0, T1 and T2 (Supplementary Material 4).

### Experienced client autonomy

Autonomy scores were analysed for 1129 (94%) and 834 (91%) parents at baseline and follow-up, respectively. At baseline, autonomy scores differed significantly between parents visiting different CJG organisations. Youth Care visitors reported the highest scores (Estimated Marginal Means (EMM) 4.13 95% CI [4.07, 4.20]), and PCH 4–18 visitors reported the lowest scores (EMM 3.82 95% CI [3.75, 3.89]) (Table 1). After 12 months, parents generally showed significantly higher autonomy scores (EMM 4.26 95% CI [4.22, 4.30]) than parents in the baseline group (EMM 4.03 95% CI [3.99, 4.07]). Moreover, portal users (EMM 4.35 95% CI [4.29, 4.41]) reported significantly more experienced autonomy than non-users (EMM 4.17 95% CI [4.11, 4.22]). The difference between PCH 4–18 visitors (EMM 4.12 95% CI [4.05, 4.26]) and Youth Care visitors (EMM 4.28 95% CI [4.21, 4.35]) was no longer significant.

**Table 1.** Client autonomy scores. Estimated marginal means (EMM) for client autonomy at baseline and follow-up, based on a general linear model. Organisation and portal use were included with fixed effects among parents, whereas age group and portal use were included with fixed effects among adolescents.

	Baseline		Follow-up		Δ
	*n*	EMM (95% CI)	*n*	EMM (95% CI)	Baseline/follow-up
Parents					
Autonomy score	1129	4.03 (3.99–4.07)	834	4.26 (4.22–4.30)	0.23
Portal use (adjusted for organisation)					
No	373	3.99 (3.93–4.05)	375	4.17 (4.11–4.22)	0.18
Yes	756	4.07 (4.02–4.12)	459	4.35 (4.29–4.41)	0.28
Organisation (adjusted for portal use)					
PCH 0–3	882	4.08 (4.04–4.11)	495	4.33 (4.29–4.36)	0.25
PCH 4–18	77	3.82 (3.75–3.89)	169	4.12 (4.05–4.26)	0.30
Youth care	170	4.13 (4.07–4.20)	170	4.28 (4.21–4.35)	0.15
Adolescents					
Autonomy score	196	3.94 (3.82–4.06)	77	4.47 (4.33–4.62)	0.53
Portal use (adjusted for age)					
No	196	3.94 (3.82–4.06)	64	4.36 (4.24–4.49)	0.42
Yes	0	NA	13	4.59 (4.32–4.86)	NA
Age group (adjusted for portal use)					
12–15 years	164	4.07 (3.99–4.14)	33	4.32 (4.11–4.52)	0.25
16–17 years	21	3.79 (3.58–4.00)	26	4.44 (4.22–4.65)	0.65
18+ years	11	3.97 (3.68–4.26)	17	4.67 (4.43–4.91)	0.70

PCH 0–3 = Preventive Child Healthcare for preschool children up to 3 years old.

PCH 4–18 = Preventive Child Healthcare for children aged 4–18 years old.

NA = Not applicable.

Autonomy scores were analysed for 192 (95%) and 77 (87%) adolescents at baseline and follow-up, respectively. At baseline, all adolescent respondents were portal non-users because they lacked access prior to introducing EPR-Youth. After 12 months, respondents showed significant higher autonomy scores (EMM 4.47 95% CI [4.33, 4.62]) than respondents in the baseline group (EMM 3.94 95% CI [3.82, 4.06]) (Table 1). No significant difference in autonomy score was found between portal users (EMM 4.59 95% CI [4.32, 4.86]) and non-users (EMM 4.36 95% CI [4.24, 4.49]). Adolescents aged 12–15 years did not experience significantly more autonomy at follow-up (EMM 4.32 95% CI [4.11, 4.52]) than at baseline (EMM 4.07 95% CI [3.99, 4.14]), as opposed to adolescents aged 16–17 years or 18 years and older.

### Professional autonomy-supportive behaviour

Overall, professionals reported no difference in autonomy-supportive behaviour when we analysed paired data of T0 (Mean 4.13 95% CI [3.99, 4.28]), T1 (Mean 4.07 95% CI [3.92, 4.21]) and T2 (Mean 4.11 95% CI [3.95, 4.28]) (Table 2). The additional univariate ANOVA, comparing all respondents at T0 (EMM 4.08 95% CI [3.94, 4.23]) with respondents completing only T2 (EMM 3.97 95% CI [3.75, 4.18]) and all respondents at T2 (EMM 4.03 95% CI [3.88, 4.18]) with respondents completing only T0 (EMM 3.98 95% CI [3.72, 4.24]), showed no difference over time either (Table 2).

**Table 2.** Professional autonomy-supportive behaviour. Means of paired data were compared over T0 (baseline), T1 (5 months after introduction of EPR-Youth) and T2 (2 years after introduction of EPR-Youth), significance was tested at the 0.05 level in a repeated measures ANOVA; estimated marginal means of unpaired data were compared in two ways between T0 (baseline) and T2 (2 years after introduction of EPR-Youth), using a general linear model. Significance was tested at the 0.05 level in an univariate ANOVA.

	T0		T1		T2	
	*n*	Mean (95% CI)	*n*	Mean (95% CI)	*n*	Mean (95% CI)
Mean professional autonomy-supportive behaviour scores, paired data (repeated measures ANOVA)						
Paired data T0-T1-T2	43	4.13 (3.99–4.28)	43	4.07 (3.92–4.21)	43	4.11 (3.95–4.28)
Estimated marginal means for professional autonomy-supportive behaviour scores, unpaired data T0 and T2 (univariate ANOVA)						
All T0 vs T2 only, adjusted for organisation	97	4.08 (3.94–4.23)			46	3.97 (3.75–4.18)
Organisation						
PCH 0–3	26	4.08 (3.87–4.30)			8	3.83 (3.34–431)
PCH 4–18	10	3.91 (3.57–4.25)			5	3.83 (3.45–4.21)
Youth care	61	4.25 (4.12–4.39)			33	4.24 (4.06–4.43)
All T2 vs T0 only, adjusted for organisation	36	3.98 (3.72–4.24)			105	4.03 (3.88–4.18)
Organisation						
PCH 0–3	3	3.75 (3.12–4.38)			31	4.11 (3.91–4.30)
PCH 4–18	7	3.95 (3.54–4.36)			8	3.72 (3.34–4.10)
Youth care	26	4.25 (4.04–4.46)			66	4.27 (4.13–4.40)

However, we found some differences in autonomy-supportive behaviour among professionals from different CJG organisations. In the second follow-up questionnaire round, youth care professionals reported engaging more frequently in autonomy-supportive behaviour (EMM 4.27 95% CI [4.13, 4.40]) than preventive school healthcare professionals (EMM 3.72 95% CI [3.34, 4.10]).

Moreover, professionals responded differently for the four elements of autonomy-supportive behaviour (Supplementary Material 5). The element ‘capability’ scored the highest: on the questions whether they asked clients what was going well and whether they asked how clients had tried to resolve a problem, 88–103 (89–96%) responded with ‘often’ or ‘always’. The element ‘network’ scored the lowest. At T2, 32 (57%) professionals reported that they often or always asked clients who they wanted to involve in their situation, whereas 14 (25%) professionals responded that they sometimes or never asked this question.

### Focus group interviews

We conducted two focus group interviews with a mix of parents (*n* = 8) and adolescents (*n* = 4) and two focus group interviews with professionals (*n* = 12). Client focus group participants represented all six municipalities, both male and female participants, with various educational levels, and used different CJG services. Professional focus group participants represented all professions and organisations at different work experience levels. All participants were native Dutch (Supplementary Material 4) and had used EPR-Youth at least once. Four main themes emerged from the thematic analysis: ownership, motivation, capability and professional-client relationship. Two relevant sub-themes emerged that were linked to capability: adolescents’ capability and balance between client autonomy and professional responsibility.

#### Elements of autonomy

##### Ownership

Parents and adolescents highly valued access to EPR-Youth. Reading a report after visiting a CJG enhanced their sense of ownership. Consequently, they were involved in their visit reports, writing comments or requesting changes. Parents and adolescents considered their right to grant access to their record as an important contributor to ownership.

“Especially as a child, you just want to have ownership, to decide who can read your record.” (Ado-lescent, 17 years)

Professionals also observed an increase in parental ownership. Parents were increasingly giving feedback on reports and planned appointments as needed, whereas professionals initiated prior appointments.

“Reports are always checked with us for accuracy. In the last report, I made some changes.” (Father, two children)

##### Motivation

Adolescents did not value client portal use for all purposes. For instance, they preferred using WhatsApp messenger instead of logging in to EPR-Youth when they wanted to ask questions or plan a new appointment. They appreciated that they could select a medium that matched their preferences.

“What do you do when you have small questions in between appointments?” “Oh yeah, most of the time I WhatsApp X” (Mother, two children, discussing with adolescent, 18 years)

##### Capability

Both parents and adolescents reported that having 24/7 access to EPR-Youth enhanced their capability, enabling them to manage their own appointments and ask questions at their convenience. Professionals reported enhanced self-management of appointments from using EPR-Youth.

“I like that I can just drop my question whenever it is convenient for me. I don’t have to plan a visit because I have plenty of other things to do. I could just describe what I saw, and based on that, we could decide what to do next.” (Mother, one child)

##### Capability adolescents

Both professionals and parents doubted the feasibility of adolescent portal access at the age of 12. They expressed concerns that young adolescents were incapable of dealing with confidential information in their record about their parents or about family circumstances. Professionals struggled to fund balance between guarding parents’ privacy, protecting young adolescents from potentially harmful information, and reporting objectively, especially in difficult situations.

“I definitely don’t want my child to read what is reported here. When she’s 12 years old, the child would be devastated if she reads what is reported here.” (Mother, two children)

“I really struggle with custody battles. I just keep thinking: How am I going to report this? I don’t think a 12-year-old kid should read about this.” (Youth care worker)

##### Client autonomy vs professional responsibility

Although both professionals and clients valued the fact that clients could become decision-makers and manage their appointments, they also expressed concerns about the counter side of autonomy. For instance, in cases of suspected child neglect, parental autonomy could pose a risk. Participants agreed that client autonomy should not be unlimited. They considered child well-being a shared responsibility between professionals and parents: when parents would not take responsibility, a professional should act upon their responsibility to protect a child.

“In our village….people who mess up don’t show up at the CJG. I know someone who has a 3-year-old boy who isn’t talking yet… If he had been visiting the CJG regularly, it could have been detected earlier. And that’s just it: that freedom can be very dangerous.” (Mother, three children)

##### Professional-client relationship

Both professionals and clients reported that using EPR-Youth contributed to a more equal relationship and collaboration between them. Collaboration evolved naturally when clients read their health records and became more involved in care processes. Moreover, the professional-client relationship was strengthened because the transparency of EPR-Youth enhanced clients’ trust in CJG professionals. Parents emphasised that professional autonomy-supportive behaviour was essential to building a relationship and collaborating on an equal basis.

“If you want people to trust you a bit more…strengthen the bond with parents…if you want to be more on the same page, then I think transparency is important too.” (Mother, two children)

“As a mother with a first child, of course, you are nervous when you visit the CJG. So, when you read afterwards, they thought you were doing a good job… that’s reassuring and makes your self-confidence grow.” (Mother, one child)

### Data integration

The outcomes of both client questionnaire and focus group interviews show that using EPR-Youth contributes to client autonomy. Moreover, the qualitative findings expand on specific elements where this contribution occurs: ownership, motivation and capability. Simultaneously, focus group interviews revealed possible limitations of autonomy for young adolescents or parents.

Clients participating in focus groups reported that professional autonomy-supportive behaviour was essential to building a relationship; thus, we expected to find a positive correlation between the extent to which professionals in an organisation reported autonomy-supportive behaviour and the extent to which clients visiting that organisation experienced autonomy. However, questionnaire outcomes did not support this assumption.

## Discussion

### General

In this study, we investigated whether using EPR-Youth in youth care and PCH contributed to experienced autonomy among adolescents and parents and whether professional autonomy-supportive behaviour added to that effect. We found that using EPR-Youth enhanced experienced autonomy among parents and adolescents, and professional autonomy-supportive behaviour was an important additional factor. Among adolescents, age also contributed to experienced autonomy.

### Experienced autonomy

Parents and adolescents experienced more client autonomy 12 months after EPR-Youth was introduced. More specifically, they felt that EPR-Youth contributed to their sense of ownership and to their capability to manage care according to their motivation. Previous research on parent-held child health records shows results comparable to those of our study. Using such records contributed to feelings of empowerment and confidence among parents, helped them make decisions for their children, and strengthened the professional-client relationship (Clendon and Dignam, 2010; Kelly et al., 2017; Lee et al., 2016; Magwood et al., 2018).

### Adolescent autonomy

No previous studies have investigated the effect of client-accessible records on adolescent autonomy.

In our study, all adolescents perceived more client autonomy over time. Adolescents aged 16 years and older, however, showed a larger increase in autonomy than those aged 12–15 years. This might be a consequence of how Dutch privacy and healthcare legislation (Dutch Ministry of Justice, 2006) supports autonomy during adolescence. Regarding autonomy, adolescents represent a specific group transitioning from childhood to maturity (Gaete, 2015). Gaining personal autonomy is part of this transition and is supported by most Western countries’ legislation (Calman et al., 2015; Essén et al., 2018; Hagström et al., 2022). At the age of 12, children supposedly have the capacities for decision-making, and simultaneously may need parental support to facilitate the process (Grootens-Wiegers et al., 2017). Moreover, parents and professionals in our study assumed that 12-year-old children would also require support dealing with sensitive information in their records. Dutch legislation anticipates both capacities and a need for support, granting shared access to medical information to both parents and adolescents aged 12–16 years (Dutch Ministry of Justice, 2006; Dutch Ministry of Public Health, 2014). Consequently, younger adolescents are encouraged to make decisions with parents or legal guardians about their care, whereas adolescents aged 16 and older have more opportunities to make their own choices. Therefore, the stronger effect among older adolescents is probably due to a combination of age-dependent growth of autonomy and changes in legislation and rights from the age of 16.

### Vulnerable groups

According to our findings, sex, educational level, native country and family constitution did not influence client autonomy. Previous research showed, however, that vulnerable groups reported more benefits from using a client portal than average because reading their records increased their understanding of the care process and helped them make decisions about their care (Benjamins et al., 2021; Jackson et al., 2014) and that vulnerable groups particularly valued involvement of family and friends (Benjamins et al., 2021; Gerard et al., 2018; Jackson et al., 2014). A possible explanation for the difference between our outcomes and earlier research is that lower-educated and clients of non-Dutch nativity were underrepresented in our study and that the last group was not represented at all in our focus groups.

### Professional autonomy-supportive behaviour

After 12 months, both portal users and non-users experienced more client autonomy, although portal users scored higher than non-users. This suggests that using EPR-Youth was not the only factor contributing to client autonomy (Benjamins et al., 2021). Focus group participants emphasised that an autonomy-supportive attitude was also an important factor, strengthening their sense of ownership and building an equal professional-client relationship. Irrespective of using client-accessible records, when professionals respect a client’s ownership over their care, support clients to use their capabilities, and follow a client’s intrinsic motivation, their behaviour stimulates client autonomy (Meinema, 2017; Ridgway et al., 2021). When autonomy-supportive behaviour is combined with client-accessible records, this combination of behaviour and technology enhances the professional-client relationship and helps persons to make better decisions about their care (ElKefi and Asan, 2021).

In line with literature and our qualitative data, we expected to find a correlation between professionals’ scores in one organisation and scores of clients visiting that organisation. This assumption was not supported by our quantitative data, perhaps because client scores could not be linked personally to their own care providers’ score.

### Strengths and limitations

With this study, we examined client autonomy from a professional and client perspective and included both parents and adolescents in the client perspective. Combining quantitative and qualitative methods has proved useful in deepening our understanding of client autonomy.

The design, tailored to be feasible in a ‘care for youth’ context, had some limitations. Inviting clients during a regular visit to a CJG location resulted in including different client groups at baseline and follow-up. Consequently, establishing a causal relation between using EPR-Youth and experienced client autonomy proved difficult.

Contrastingly, the paired-measures design for the professional questionnaire would have allowed us to establish a causal relationship between using EPR-Youth and an autonomy-supportive attitude. However, the low response rate at T1, due to increased workload when the COVID-19 pandemic started, drastically reduced the possibility to pair data over three measurements. Therefore, we had to adapt our design and analysis plan, and re-invited all CJG professionals to complete the questionnaire at T2. Combining the analyses of paired and unpaired data, the analyses of unpaired data confirmed the analysis of paired data, leading to a stronger conclusion.

The small number of adolescent respondents and the underrepresentation of vulnerable groups diminished generalisability of our outcomes. Moreover, our study was conducted in a rural area with a relatively low educated population and a small minority with a migrant background.

More research is required, with larger numbers of adolescents and a broader representation of all population groups, to generate more generalisable outcomes in this specific target group. Furthermore, paired data are needed to establish a causal relationship between using client-accessible records and experienced autonomy.

### Implications for practice

Our findings support the use of client-accessible records as a tool to enhance autonomy among parents and adolescents. However, when organisations implement client-accessible records with the aim of strengthening autonomy in this target group, two issues need addressing. First, professionals should adopt an autonomy-supportive attitude. Second, the phase between the age of 12 and 16, when parents and adolescents both have access rights, deserves attention. In this phase, adolescents should be encouraged to increasingly exercise their autonomy, whereas parents should support their children in this process and gradually step back.

## Conclusion

Our findings showed that using EPR-Youth increased perceived autonomy among parents and adolescents, contributing specifically to ownership, motivation and capability. This contribution was stronger among adolescents aged 16 and older, probably due to different legal rights. Among younger adolescents, the balance between growing autonomy and need for parental support requires attention. Over time, no change in professional autonomy-supportive behaviour was found, although differences were found between professionals from different organisations. Clients considered professional autonomy-supporting behaviour essential to benefit from using EPR-Youth. Therefore, organisations implementing client-accessible records should address professional attitude. Follow-up research with paired data is needed to confirm that the found association between using EPR-Youth and perceived client autonomy is a causal relationship.

## Supplemental Material

Supplemental Material - Effect of using client-accessible youth health records on experienced autonomy among parents and adolescents in preventive child healthcare and youth care: A mixed methods intervention study Supplemental Material for Effect of using client-accessible youth health records on experienced autonomy among parents and adolescents in preventive child healthcare and youth care: A mixed methods intervention study by Janine Benjamins, Emely de Vet, Gerlinde Jordaan and Annemien Haveman-Nies in Journal of Child Health Care.

## Appendix

### Abbreviations

CJG: Centre for Youth and Family
EPR-Youth: Electronic Patient Record in Care for Youth
PCH: Preventive Child HealthCare
