# Supplementary material for: Effect of using client-accessible youth health records on experienced autonomy among parents and adolescents in preventive child healthcare and youth care: A mixed methods intervention study
Source: J Child Health Care. 2023 May 25;29(1):79–96. doi: 10.1177/13674935231177782 (PMC11874616; doi:10.1177/13674935231177782)

Supplementary Material 1: client's questionnaire, experienced autonomy

With the following 5 questions, three quadrants of the Movisie model for stimulating client's autonomy were operationalized: ownership, capability and motivation. The same questions were used for parents and adolescents.

1. If you have questions, do we build further on the things you already know, the capacities you have and the things you already do?
   Answering categories: very angry smiley (1) to very happy smiley (5)

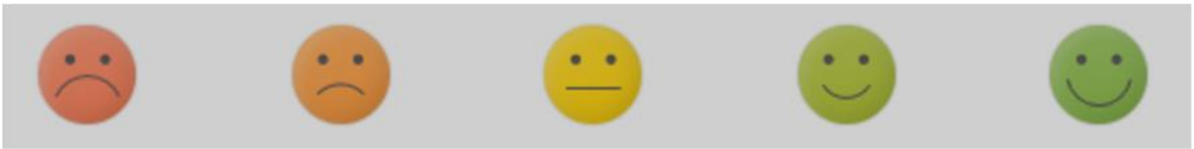

2. If you have questions, do we allow you to choose which plan and solution fits with you/your child/your family?
   1. This never happens
   2. This happens occasionally
   3. This happens as often as not
   4. This happens often
   5. This always happens

3. Do you appreciate the possibility to read and write in your child's/ your record?

Smiley faces from red (1) to dark green (5)

4. Does our advice usually match with your needs?

Smiley faces from red (1) to dark green (5)

5. Continue on your own strength: Do our conversations help you/your family to (at a certain point) move on independently?
   1. They never help
   2. They almost never help
   3. They help as often as not
   4. They often help
   5. They always help

6. Portal use: have you ever accessed your client portal/ the client portal of your child?

1. Yes, I have
2. I tried, but it did not work (Analyzed as ‘no’)
3. No, I have not

Supplementary Material 2: professional's questionnaire, contribution to client’s autonomy.

Every quadrant of the Movisie model was operationalized in two statements. Professionals were responding on a 5-point Likert scale, with the answering options as formulated below. To match high scores with positive opinions, all scores from the professional questionnaires were reversed.

Capability:

1. In conversations with parents/adolescents I ask what is going well.
2. In conversations with parents/adolescents I ask what they have already tried to resolve their problem.

Network:

3. In conversations with parents/adolescents I ask who else is concerned about the wellbeing of this adolescent/child and this family.
4. In conversations with parents/adolescents I ask who they want to involve in their situation.

Motivation

5. In solving an issue, I discuss with parents/adolescents what their values are for ‘a good life’.
6. In solving an issue, it is most important what the parent/adolescent wants to achieve.

Ownership

7. The parent/adolescent usually decides what he/she wants to keep and what needs to change.
8. I only contribute to the solution of an issue when the parent/adolescent indicates that this is necessary.

Answering options questions 1-5:

1 = always

2 = often

3 = 50/50

4 = sometimes

5 = never

Answering options questions 6-8:

1 = totally agree

2 = somewhat agree

3 = neutral

4 = somewhat disagree

5 = totally disagree

Supplementary Material 3: semi-structured focus group interview guide

Interview scope:

- Professionals – How do professionals experience the impact of using EPR-Youth on client autonomy?
    - How do professionals feel about a client-accessible health record; what positive and negative connotations do they have?
- Parents/adolescents – How do parents and adolescents experience the impact of using EPR-Youth and its client portal on their autonomy?
    - How do parents and adolescents feel about a client-accessible health record; what positive and negative connotations do they have?

Topic list:

- In general: what experiences can you describe, using EPR-Youth or the client portal?
    - ⇒ Positive, what is working well; in what way is it helpful?
    - ⇒ Negative, what could work better, what is not helping? Do you have any suggestions?
    - ⇒ Clients: were you aware of the existence of a client-accessible health record before you were invited to this interview? Have you logged in to the client portal?
    - ⇒ Clients: what is your opinion on the possibility to read all registrations?
    - ⇒ Clients: Has the care process or the communication with your caretaker altered in any way because of the use of EPR-Youth?
- Autonomy
    - ⇒ In what way does the use of EPR-Youth contribute to clients' autonomy?
        - ◊ Clients (explanation): with the term 'contributing to autonomy' we mean that you are in control of your own care process and make joint decisions with your care provider. We want to create care plans that fit with your personal needs and identity. We want to support that with the development of EPR-Youth. Do you experience that EPR-Youth strengthens your autonomy? What does it contribute to your autonomy that you can:
            - Read everything we write
            - Ask questions in the portal
            - Check and manage your appointments
            - See in the view log which professional has been working in your EPR-Youth
            - Comment on our registrations
            - Add your own information, care plan and hospital letters to EPR-Youth
    - ⇒ What is your role in stimulating clients' autonomy?

◊ Professionals: informing clients, collaboration, explain view log/planning/questions, discuss registrations, grant access

◊ Professionals: do you use all functionalities of EPR-Youth?

◊ Clients: Do you use all functionalities of EPR-Youth? If not: why not? Would you want to use them? What would you need to use them?

⇒ What is needed to enhance autonomy?

◊ What could you do?

◊ What do you need from managers and staff?

◊ What needs to be adapted in EPR-Youth?

Supplementary Material 4: Respondent Characteristics

A: Client questionnaire
B: Professional questionnaire
C: Focus group participants

Table A: Characteristics of parents and adolescents who completed the client questionnaire, at baseline and follow-up. Absolute numbers are given, and percentages between brackets. We distinguished three educational levels for parents and two for adolescents. For the variables educational level, sex, and native country, distribution within the source population is shown in percentages.

	Parents		Adolescents		North-Veluwe
	Baseline n=1202 (%)	Follow-up n=914 (%)	Baseline n=202 (%)	Follow-up n=89 (%)	%
**Educational level**					
Low	150 (12·5)[a]	63 (7·4)[a]	138 (71·1)[a]	40 (56·3)[a]	30%
Middle	543 (45·2)[a]	414 (43·3)[a]	NA	NA	41%
High	509 (42·3)[a]	380 (38·9)[a]	56 (28·9)[a]	31 (43·7)[a]	29%
Missing	0	57	8	18	
**Sex**					
Male	126 (10·5)	97 (11·3)	95 (47·5)[a]	11 (14·3)[a]	50%
Female	1076 (89·5)	760 (88·7)	105 (52·5)[a]	66 (85·7)[a]	50%
Missing	0	57	2	12	
**Native country**					
The Netherlands	NA	791 (96·2)	NA	72 (97·3)	92%
Other	NA	31 (3·8)	NA	3 (2·7)	8%
Missing	NA	92	NA	14	
**Family composition**					
2-Parent family	1017 (84·6)[a]	768 (90·0)[a]	147 (73·1)[a]	31 (41·3)[a]	-
Other situation	185 (15·4)[a]	85 (10·0)[a]	54 (26·9)[a]	44 (58·7)[a]	-
Missing	0	61	1	14	
**Age children**					
Children 0-3 y	949 (79·0)[a]	521 (61·1)[a]	NA	NA	-
Children 4-11 y	168 (14·0)[a]	255 (29·9)[a]	NA	NA	-
Children 12+	85 (7·1)[a]	77 (9·0)[a]	NA	NA	-
Missing	0	61	NA	NA	
**Age adolescents**					
12-15 y	NA	NA	169 (84·1)[a]	33 (42·9)[a]	-
16/17 y	NA	NA	21 (10·4)[a]	26 (35·1)[a]	-
18+ y	NA	NA	11 (5·5)[a]	17 (22·1)[a]	-
Missing	NA	NA	1	12	
**Organization**					
PCH 0-3 yrs	891 (77·6)[a]	495 (57·5)[a]	NA	NA	-
PCH 4-18 yrs	86 (7·5)[a]	187 (21.7)[a]	119 (68·4)[a]	21 (32·3)[a]	-
Youth care	171 (14·9)[a]	179 (20·8)[a]	55 (31·6)[a]	44 (67·7)[a]	-
Unknown/missing	54	53	28	24	
**Portal use**					
No	415 (34·5)[a]	421 (46·2)[a]	202 (100)[a]	73 (83·9)[a]	-
Yes	787 (65·5)[a]	490 (53·8)[a]	NA[a]	14 (16·1)[a]	-
Missing	0	3	0	2	

[a]: Significant difference between baseline and follow-up group, as tested with Pearson χ^2, $p<0·001$.
NA = not applicable

PCH 0-3 = preventive child healthcare for children up to 3 years old
PCH 4-18 = preventive child healthcare for children aged 4 to 18 years old.

Table B: Characteristics of professional questionnaire respondents at baseline (T0) and two follow-up moments (T1 and T2, five and 24 months after introduction of EPR-Youth). Percentages of the total number of respondents are represented between brackets. Differences in respondent characteristics between T0, T1 and T2 were tested with Pearson Chi-square, at a 0.05 significance level.

	T0 n=100 (%)	T1 n=57 (%)	T2 n=110 (%)	Pearson χ^2	2-sided p-value
**Sex**					
male	9 (9·0)	4 (7·0)	9 (8·2)	0·19	0·91
female	91 (91·0)	53 (93·0)	101 (91·8)		
**Working experience**					
0-10 years	35 (35·0)	18 (31·6)	50 (45·5)	6·85	0·14
10-20 years	35 (35·0)	18 (31·6)	38 (34·5)		
>20 years	30 (30·0)	21 (36·8)	22 (20·0)		
**Profession**					
Behavioral expert	4 (4·0)	1 (1·8)	8 (7·3)	3·46	0·90
PCH Doctor	11 (11·0)	7 (12·3)	10 (9·1)		
Youth Care Worker	58 (58·0)	31 (54·4)	62 (56·4)		
PCH Nurse	25 (25·0)	17 (29·8)	28 (25·5)		
PCH Speech therapist	2 (2·0)	1 (1·8)	2 (1·8)		
**Organisation**					
PCH 4-18	12 (12·0)	4 (7·0)	12 (10·9)	2·78	0·60
PCH 0-3	26 (26·0)	21 (36·8)	30 (27·3)		
Youth Care	62 (62·0)	32 (56·1)	68 (61·8)		

PCH = preventive child healthcare
PCH 0-3 = preventive child healthcare for children up to 3 years old
PCH 4-18 = preventive child healthcare for children aged 4 to 18 years old.

Table C: Characteristics of focus group participants, separately for clients (n=12) and professionals (n=12). Percentages of the total number of participants from each group are represented between brackets.

	**Clients**	n=12 (%)
**Parent or adolescent**	Parent	8 (67)
	Adolescent	4 (33)
**Sex**	Male	4 (33)
	Female	8 (67)
**Educational level**	High	4 (33)
	Middle	5 (42)
	Low	3 (25)
**Native country**	the Netherlands	12 (100)
	Other	0 (0)
**PCH/ Youth care**	PCH	4 (33)
	Youth care	8 (67)
	**Professionals**	n=12 (%)
**Sex**	Male	1 (8)
	Female	11 (92)
**Working experience**	Less than 5 years	4 (33)
	5 to 10 years	2 (17)
	More than 10 years	6 (50)
**Profession**	Doctor	2 (17)
	Nurse	3 (25)
	Behavioral scientist	1 (8)
	Youth worker	3 (25)
	Administrative	2 (17)
	Screener	1 (8)
**Organization**	Youth care	4 (33)
	PCH 0-3	6 (50)
	PCH 4-18	2 (17)

PCH = preventive child healthcare
PCH 0-3 = preventive child healthcare for children up to 3 years old
PCH 4-18 = preventive child healthcare for children aged 4 to 18 years old.

supplementary_material_5-supportive_behaviour

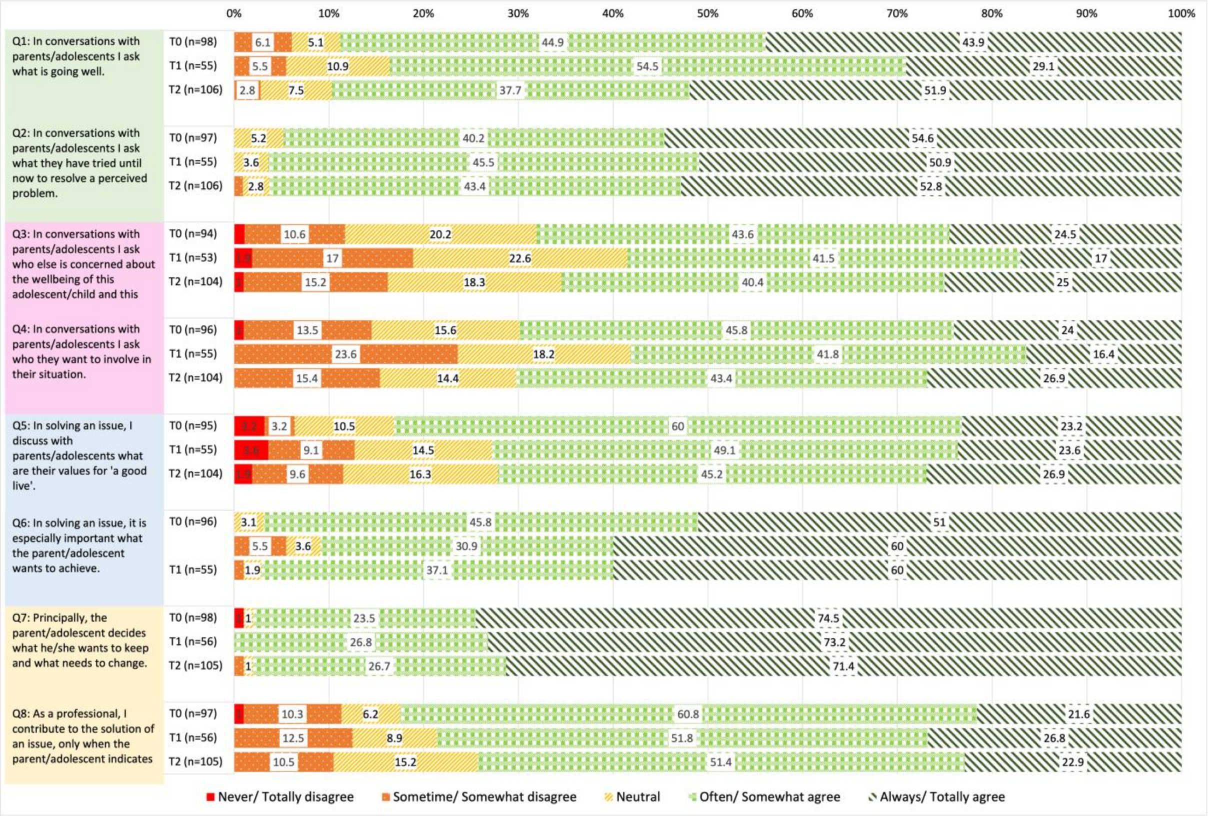

Supplement: Supplemental Material - Effect of using client-accessible youth health records on experienced autonomy among parents and adolescents in preventive child healthcare and youth care: A mixed methods intervention study [file sj-pdf-1-chc-10.1177_13674935231177782.pdf]
